# A realist evaluation of community-based participatory research: partnership synergy, trust building and related ripple effects

**DOI:** 10.1186/s12889-015-1949-1

**Published:** 2015-07-30

**Authors:** Justin Jagosh, Paula L. Bush, Jon Salsberg, Ann C. Macaulay, Trish Greenhalgh, Geoff Wong, Margaret Cargo, Lawrence W. Green, Carol P. Herbert, Pierre Pluye

**Affiliations:** Centre for the Advancement of Realist Evaluation and Synthesis, Waterhouse Building, Block B, Brownlow Street, Liverpool, L69 3GL UK; Department of Family Medicine, 5858 Ch. de la Cote-des-Neiges, 3rd floor, Montreal, QC H3S 1Z1 Canada; Nuffield Department of Primary Care Health Sciences, University of Oxford, New Radcliffe House, Radcliffe Observatory Quarter, Woodstock Road, Oxford, OX2 6GG UK; University of Southern Australia, School of Population Health, North Terrace, Adelaide, South Australia 5000 Australia; Department of Epidemiology and Biostatistics, University of California at San Francisco, Box 0981, UCSF, San Francisco, CA 94143-0981 USA; Department of Family Medicine, Schulich School of Medicine & Dentistry, University of Western Ontario, London, ON Canada

**Keywords:** Community-based participatory research, Public health, Realist synthesis, Realist analysis, Sustainability, Partnership synergy, Trust, Spin-off projects, Systemic transformations, Ripple effect

## Abstract

**Background:**

Community-Based Participatory Research (CBPR) is an approach in which researchers and community stakeholders form equitable partnerships to tackle issues related to community health improvement and knowledge production. Our 2012 realist review of CBPR outcomes reported long-term effects that were touched upon but not fully explained in the retained literature. To further explore such effects, interviews were conducted with academic and community partners of partnerships retained in the review. Realist methodology was used to increase the understanding of what supports partnership synergy in successful long-term CBPR partnerships, and to further document how equitable partnerships can result in numerous benefits including the sustainability of relationships, research and solutions.

**Methods:**

Building on our previous realist review of CBPR, we contacted the authors of longitudinal studies of academic-community partnerships retained in the review. Twenty-four participants (community members and researchers) from 11 partnerships were interviewed. Realist logic of analysis was used, involving middle-range theory, context-mechanism-outcome configuration (CMOcs) and the concept of the ‘ripple effect’.

**Results:**

The analysis supports the central importance of developing and strengthening partnership synergy through trust. The ripple effect concept in conjunction with CMOcs showed that a sense of trust amongst CBPR members was a prominent mechanism leading to partnership sustainability. This in turn resulted in population-level outcomes including: (a) sustaining collaborative efforts toward health improvement; (b) generating spin-off projects; and (c) achieving systemic transformations.

**Conclusion:**

These results add to other studies on improving the science of CBPR in partnerships with a high level of power-sharing and co-governance. Our results suggest sustaining CBPR and achieving unanticipated benefits likely depend on trust-related mechanisms and a continuing commitment to power-sharing. These findings have implications for building successful CBPR partnerships to address challenging public health problems and the complex assessment of outcomes.

**Electronic supplementary material:**

The online version of this article (doi:10.1186/s12889-015-1949-1) contains supplementary material, which is available to authorized users.

## Background

Community Based Participatory Research (CBPR) is an approach to research in which researchers and community stakeholders (both individuals and organizations) form equitable partnerships and co-construct research for the mutual and complementary goals of community health improvement and knowledge production [1–5]. This is an increasingly popular approach across academic, governmental and philanthropic domains [6], which raises the need to better understand and develop the science of CBPR assessment [7–11]. Work in this area is growing, and includes theorizing participation and conceptual modeling [1, 12–16] and operationalizing key CBPR assessment constructs (*e.g*., trust, capacity & readiness, participation, relational dynamics, *etc*.) [8, 9, 17, 18].

We previously undertook a realist review of the benefits and implications of CBPR, focusing on how outcomes are produced through partnerships over time. Previous publications from that review addressed the rationale for using realist methodology to assess CBPR [19], the review protocol [20], overall findings [21], CBPR using quantitative methodologies [22] and critical reflections on the realist review experience in the context of CBPR [23]. A key finding was that CBPR in projects genuinely oriented to shared decision-making and co-governance yielded solutions to research barriers and created benefits for individuals, communities, institutions, and policy development [21, 22].

Our previous analyses documented that CBPR supports (a) the production of culturally and logistically appropriate research; (b) the capacity to recruit participants to projects and interventions; (c) the capacity building of academic and community partners; (d) productive conflict resolution and negotiation processes; (e) the accumulation of partnership synergy, which increases the quality of outputs and outcomes over time; (f) the capacity to sustain project goals beyond funded time frames and during gaps in external funding; and (g) the generation of systemic changes and new unanticipated projects and activity.

To explore CBPR benefits further and because we believed that there were likely experiences of both academic and community partners not captured in journal publications, the lead author (JJ) received a Canadian Institutes of Health Research (CIHR) postdoctoral fellowship to interview academic and community partners of CBPR partnerships retained in the review.

## Methodology and methods: realist evaluation

This study used realist evaluation methodology [24] informed by a realist synthesis [25]. It is based on qualitative research methods for data collection [26]. A central tenet of realist methodology is that programs work differently in different contexts – hence a community partnership that achieves ‘success’ in one setting may ‘fail’ (or only partially succeed) in another setting, because the mechanisms needed for success are triggered to different degrees in different contexts. A second tenet is that for social programs, mechanisms are the cognitive or affective responses of participants to resources offered [27]. Thus the realist methodology is well suited to the study of CBPR, which can be understood, from an ecological perspective, as a multiple intervention strategies implemented in diverse community contexts [25, 28] dependant on the dynamics of relationships among all stakeholders.

Realist methodology begins by explicating the underlying assumptions or candidate middle-range theories [29] about the mechanisms by which programs (or components of programs) might work. Candidate middle –range theories are then used to focus the research questions and develop data collection protocols. A range of data (which may be qualitative or quantitative, and typically a combination of both) are collected and then tested against the candidate theories through a heuristic process of constructing, exploring, and refining context-mechanism-outcome (CMO) configurations. The final research product from realist methodology is not a statement of ‘effect size’ (since the same program will have different effects in different contexts), but a refinement of middle-range theory [29] that addresses (some or all of) the questions: what works for whom, under what circumstances, why and how? Evidence can include primary outcome data, but also program and setting descriptions that portray contextual elements as well as interpretation of outcomes by study authors. Further detail about realist methodology can be found elsewhere [24, 27]. Table 1 provides definitions of realist concepts.

**Table 1:** Definition of terms

**Fig. 1:**
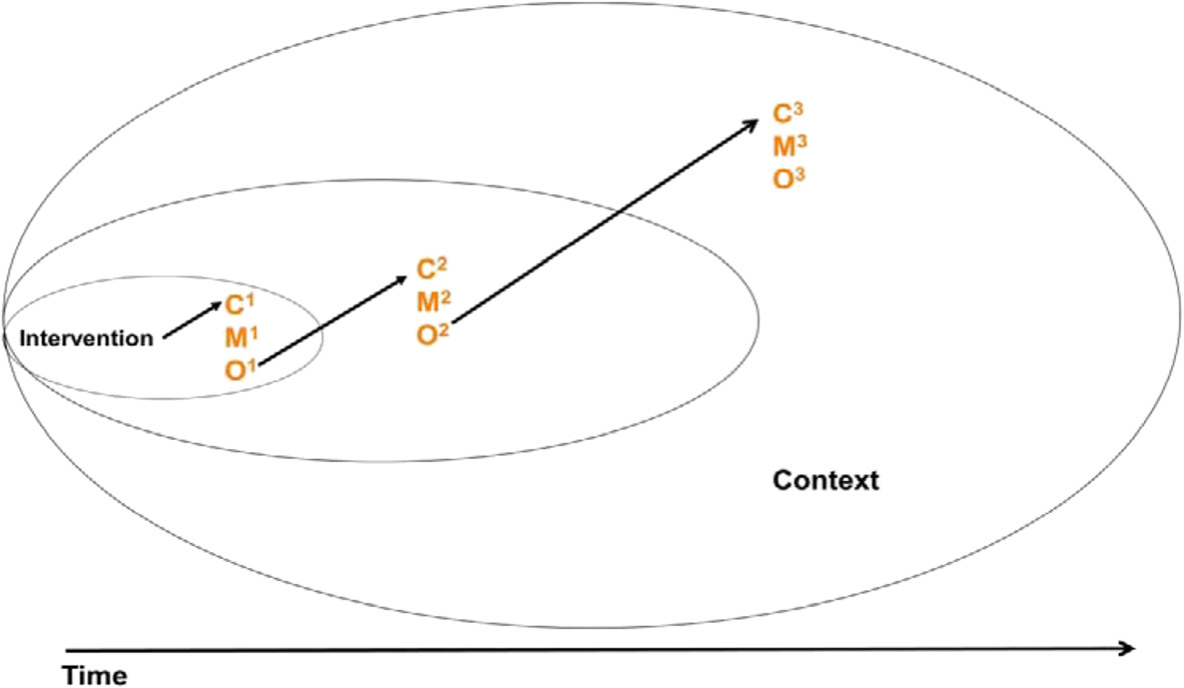
Linked context-mechanism-outcome configurations depicting the ripple effect

Realist methodology. A theory driven, interpretative approach to uncovering underlying middle-range theories (or logics) driving interventions and their multiple components, as well as illuminating the contextual factors that influence mechanisms of change to produce outcomes.
Middle-range theory (MRT): an implicit or explicit explanatory theory that can be used to explain specific elements of programs or how program logic manifests in implementation. “Middle-range” means that it can be tested with the observable data and is not abstract to the point of addressing larger social or cultural forces (*i.e.*, grand theories). MRT is sought at the outset and examined iteratively throughout the review.
Context-mechanism-outcome (CMO) configurations: CMO configuring is a heuristic used to generate causative explanations about outcomes in the observed data. A CMO configuration may be about the whole program or only to certain aspects. One CMO may be embedded in another or configured in a series (ripple effect in which the outcome of one CMO becomes the context for the next in the chain of implementation steps). Configuring CMOs is a basis for generating and/or refining the theory that becomes the final product of the review.
Context: Context often pertains to the “backdrop” of programs and research. For example, in our review of CBPR, it covers the conditions connected to the development of research partnerships. As these conditions change over time, the context may reflect aspects of those changes while the program is implemented. Examples of context include cultural norms and history of the community in which a program is implemented, the nature and scope of existing social networks, or built program infrastructure. They can also be trust-building processes, geographic location (*e.g.*, rural or urban), types of funding sources, and other opportunities or constraints.
Mechanism: the intended or unintended resources created by an intervention and the response to those resources (cognitive, emotional, motivational etc) by participants. Mechanisms can pertain to why participants choose (or choose not) to participate in interventions or internalize health knowledge or behavior change from the intervention. It may also be applied to other ‘actors’ such as implementers and staff. Mechanisms are not synonymous with strategies (*e.g.*, a strategy may be an intended plan of action, whereas a mechanism involves the resource create by the strategy + the participants’ reaction or response to the intentional offer of incentives, disciplinary actions, or other resources).
Outcomes and effects: Our interest in evaluating CBPR outcomes is not only in assessing intended outcomes (did the project succeed against the criteria it set itself at the outset), but also all the intermediate outcomes as well as unplanned and/or unexpected impacts, of which we have noted many. These are important because unplanned outcomes can sometimes have a greater influence on the determinants of health for a community than the more narrowly focussed outcome goals of projects. Furthermore, unintended impacts may have ‘ripple effects’ [42] in that they lead to new effects which then lead to more effects, thus changing the context of research overtime. The realist methodology used here is particularly suited to capturing these ripple effects in linked context-mechanism-outcome (CMO) configurations, depicted in Fig. 1.

### Sample

With ethics approval by McGill University, Faculty of Medicine Institutional Review Board, lead academic author(s) of the 23 partnerships retained in the prior review [21] were sent an invitational letter through email to participate in an interview. They were asked to recommend community partners for interviewing as well. On contact, three partnerships were discovered to be no longer active. Of the remaining 20 partnerships, seven did not respond to an email request and two declined the invitation. Partners from the remaining eleven partnerships (24 participants–14 academic and 10 community members) participated across numerous interviews and one focus group (See Additional file 1 for details). All interviews were conducted in person or by telephone by lead author (JJ). The focus group was conducted in person. A semi-structured interview guide was used to anchor the interview process (see Additional file 2). All interviews were audio recorded and transcribed.

### Analysis of interviews

Interviews were analyzed using CMO configurations which were drafted by JJ and confirmed by PLB before being discussed and refined by all other authors. This sequential feedback approach was chosen based on limited resources available for this study and because the sample scope remained limited to the 2012 review, which involved two-person independent coding. Through multiple rounds of feedback and iteration, the CMO configurations were debated and refined, along with new theoretical propositions about the effects of participation. In the findings section below, CMO configurations are linked to the clearest quotations that illustrate our theoretical propositions about trust building and sustainability arising from CBPR.

### Middle-range theory: the ripple effect of trust building in partnership synergy

With realist methodology, middle-range theory (MRT) [29] is used to explicate the underlying logic of programs, which is then tested against the evidence in various ways, using context-mechanism-outcome configurations [25] (see definitions in Table 1). Partnership synergy theory [30] was the MRT identified and developed in our CBPR realist review [21, 23]. Partnership synergy theory holds that the fair and equitable combining of skills and resources of multiple stakeholders increases the facility of research processes and achievability of results, especially over the course of time [30]. Here, we expounded the theory of partnership synergy by showing how context-mechanism-outcome configurations can be linked to each other - with the outcome of one phase of a project becoming an aspect of context for the next phase ([21] p. 329). Thus, we linked CMOcs (using: C^1^M^1^O^1^ -- > C^2^M^2^O^2^) with the concept of ‘the ripple effect’ [31] (see Fig. 1).

The ripple effect concept is premised on the idea that CBPR activity is a series of ‘events in the history of a system, leading to the evolution of new structures of interaction and new shared meanings’ p. 267 [32]. The ripple effect, in conjunction with CMO configurations, served as a framework to better understand how partnership activities accrue in stages, with the outcomes of one stage of the partnership life course informing or transforming the context for subsequent stages. As with the prior review we still view partnership synergy as the middle-range theory, but moving it forward, we theorize that trust is a foundational element of partnership synergy building. It is a commitment to building and maintaining trusting relationships over the long-term that produces an increase of synergy over time, resulting in the longitudinal outcomes that we see in terms of sustainability. Realist evaluation acknowledges ‘ontological depth’, [24]. For us, this means that what manifests in terms of CBPR process and outcomes can be understood in terms of layers of reality. At the empirical level are commonly observed outcomes, at a deeper layer we have theorized the underpinnings of such outcome in terms of the creation of partnership synergy, and at a deeper layer still are issues of trust amongst members. It is through the theorizing of CBPR through partnership synergy and trust that longitudinal outcomes come into view. Thus the role of our MRT is to say that that partnership synergy is built on trust in academic-community relationships and is a key process in sustaining partnerships in the long-term and producing the extensive array of CBPR effects over time. These effects are detailed in the findings section below, and include sustainability, spin-off projects and systemic transformations.

### Findings

By using the ripple effect concept with context-mechanism-outcome configuration (CMOc), trust was at times configured as an aspect of context (*i.e*. trust/mistrust as a *precondition* or potential *resource*), other times as a mechanism (*i.e. how* stakeholders responded to partnership activities) and was also an outcome (*i.e*. the *result* of partnership activities) in dynamically changing partnerships over time. Trust as context, mechanism and outcome in partnerships generated longer-term outcomes related to sustainability, spin-off project and systemic transformations. The findings presented below are organized in two sections: (1) Dynamics of trust and (2) Longer-term outcomes including sustainability, spin-off projects and systemic transformations.

#### Trust

**Fig. 2:**
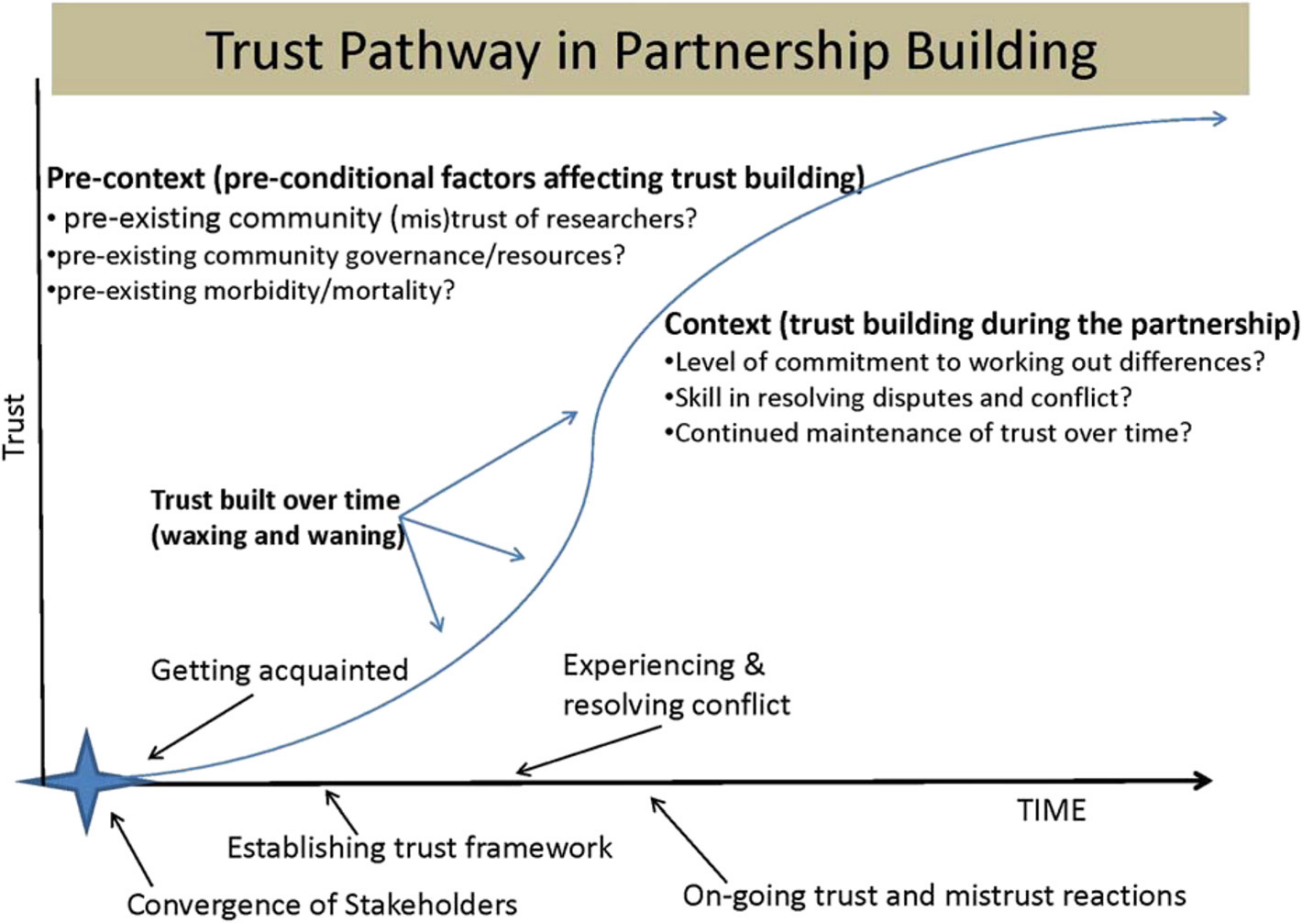
The trust pathway in partnership building

The quotations in this section illustrate the complexity of building and maintaining trusting relations for partnership members as well as the role of history and pre-context. The trust analysis is depicted visually in Fig. 2, which shows community history and other issues interacting with the process of building and maintaining trust. As one interviewee of a long standing partnership states, “…*we’re learning…that trust is [not only] built but must be maintained…every time a resource decision is made, trust comes up. …If you continually make resource decisions that are making people distrustful it’s not going to work”.* (Partnership C – academic stakeholder)

Another academic partner, working on multiple community projects, suggested that a healthy mistrust by communities toward outsider research interests, in early stages of partnership development, is a beneficial context for establishing trust in the long run:

Partnership B – Academic stakeholder: “*The community people came to the table saying, ‘…I have an earned scepticism that I’m holding with me’. And I think that’s one of the things that made it such a good strong partnership. We have another partnership [in another neighborhood], which is a…wonderful community. So they are like ‘you guys [academics] are great! this is great!’…but in terms of the process of how research gets done…because they have no level of defensiveness, and no negative experiences, they could easily be taken advantage of by people who don’t really care”.*

##### CMO configuration

The first community had prior experience with research (context) and a savvy resistance to being exploited (mechanism), which led to the establishment of trustworthy partnership relationships based on negotiation and equity (outcome). The second community did not have previous experience with research (context) and brought a naïve sense of trust and enthusiasm (mechanism) to the partnership, increasing their risk of being exploited by the research process (potential outcome) or running into unforeseen conflicts in subsequent stages, hampering productivity or partnership relations (potential outcome).

Trust was a response that was not only tested over time, but was gained through association or reputation. As the following example indicates, based on community-based networks and coherence, trust of an academic partner by one community group created a feeling of ‘trust by proxy’ by other community stakeholders:

Partnership F – Academic stakeholder: “*I attended their…* [33] *meetings and I think they were surprised that I just kept showing up. I would say, ‘hey there, me again, the doctor, but I don’t have anything and I don’t know anything’. And I did that for three years. After my residency…I said ‘ok guys, here I am. Now I can do stuff’.… they said, ‘we have this sister agency on the South side that has a whole bunch of people working on asthma. You should work with them’. So all it took was a phone call from one director to the other director saying that this doctor is going to come meet with you guys, she is good; you can trust her. So with that, they just welcomed me in.*

##### CMO configuration

There was no prior partnership relationship between the community and academic researchers (context). The academic partner demonstrated time commitment and humility over three-years and the community partners came to trust this academic partner (mechanism). Due to community networks (context) and the endorsement of the academic by one community group, the academic partner was deemed to be a credible person by others (mechanism) and began an immediate productive working relationship with an associated community agency (outcome).

Issues of trust were addressed also through the perceived importance of the research. When community partners felt invested in the need for community-based research, sincere attempts were made to improve trust and to ensure productive working relationships. The following quotation shows how a pre-conditional mistrust of outsiders by one community partner interacted with trust-building efforts overall, and the general sense of need for the research being proposed (cancer prevention and awareness):

Partnership D– Academic stakeholder “*at a community advisory board meeting [one person] talked about how I’m just another white researcher who’s coming in to rearrange people’s furniture and it was very violent. I literally felt like someone had a sword going through my gut…and this person later went on try to get me fired from the university. But the board got [covered] my back… not that people are in love with me, but people have in this project the goal of what we are working on. And that really trumps any kind of crap that gets tossed around… it’s really because there is such a strong focus on what we’re up to…what we want to have happen”.*

##### CMO configuration

A degree of resistance or hostility to outside researchers existed within the community, in part because of the historical exploitation in the name of research (context). The focus on relevant health issues for the community (*e.g.*, cervical cancer) overrode fears of repeating the history of exploitation (mechanism). Community members defended the academic member based on the research focus and the felt need to proceed with research development (outcome).

The following quotation shows how building and maintaining trust over time increased productivity in the long-run and created community environments that were conducive to research:

Partnership C – Academic stakeholder *“So, when you finish the project you may no longer want to do that project with that particular priority, but, now you got, you know, logistics - you’ve got mechanisms and trust. And working with community organizations…they are already in the game as advocates for their community [so] they will stay in the game…Because once they know that now the group is genuine; the group is effective; they will stay in the game with much less resources”.*

##### CMO configuration

The partnership had a long standing history of successful relationship building and trust among members (context). Partners felt committed to sustaining the partnership and working on new projects (mechanism). With a long-standing relationship, the coalition was able to sustain their efforts, and achieve goals with fewer resources (outcome).

#### Sustainability, spin-off projects and systemic transformations

**Fig. 3:**
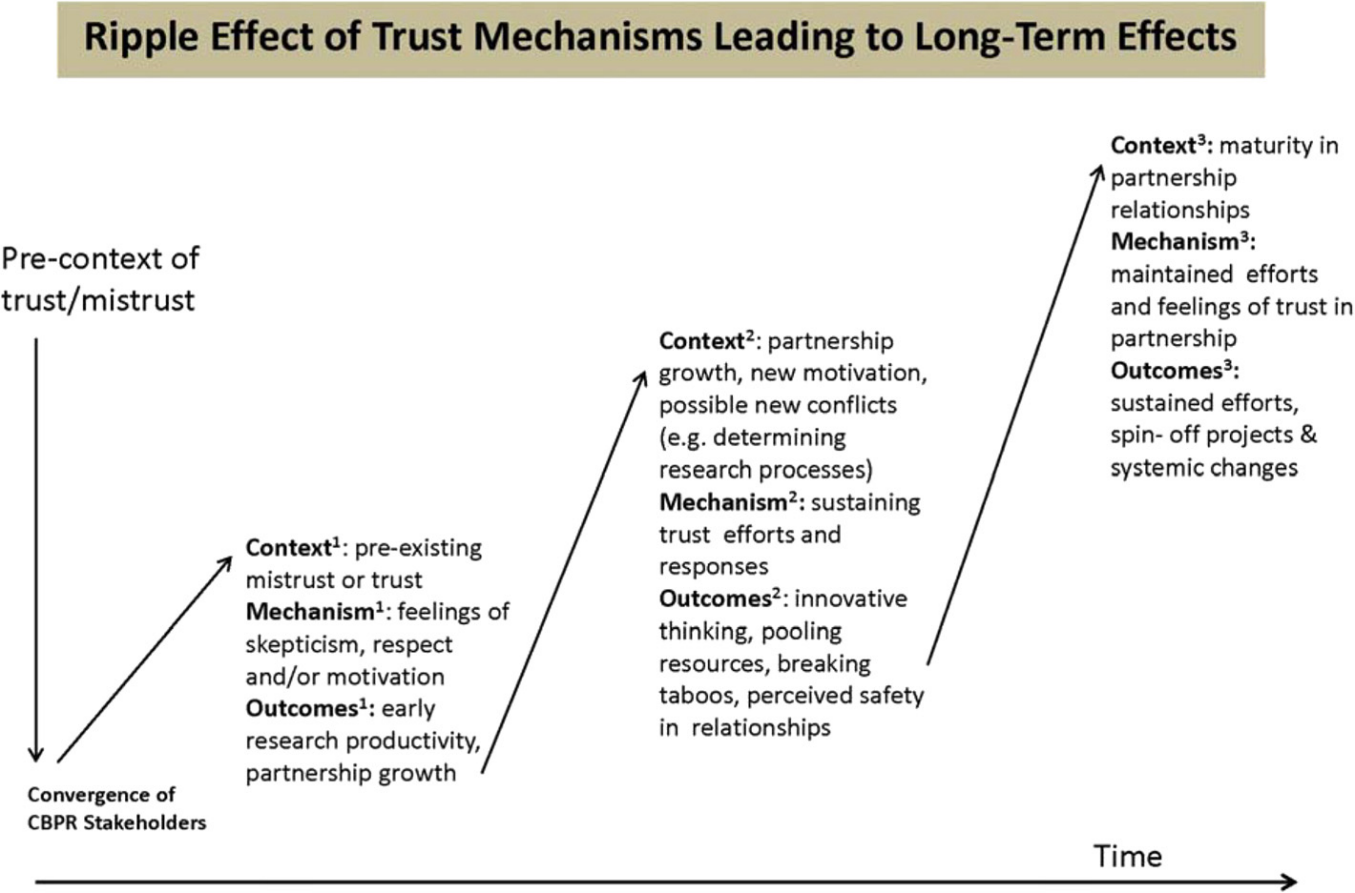
Ripple effect of trust mechanisms leading to long-term effects

As partnerships built and maintained trust over time, a host of outcomes was seen as arising from their activities. These impacts have been categorized as sustainability efforts, spin-off projects and systemic transformations. Spin-off projects were defined as unanticipated projects that arose through intentional CBPR activities, and systemic transformations meant activities that transformed elements of the physical, cultural, institutional, or policy context leading to improved health or health services outcomes. See Fig. 3 for a composite sketch of these outcomes.

##### Spin-off projects

With successful research activities, partnerships reported creating new, unanticipated projects and activities. This was credited to the trust built and maintained over time, and the expanded need for services. One community partner described how the partnership which had achieved success in raising awareness to counter female reproductive cancers, felt the need to sustain the partnership when the men in the community requested their help:

Partnership D – Community stakeholder *‘I didn’t expect that we would become a non-profit. We were coming to the close of the project and it just seemed…we just had to continue our services. And we began to see that the men are just getting alerted to this too and there’s just so much more that we could do so we had to become a non-profit.’*

##### CMO configuration

The research partnership had been in productive operation for many years (context^1^) and had gained profile and status within the community. The community saw value to the work that was being done by the coalition (mechanism^1^) and approached them with ideas for other projects (outcome^1^). This prompted the coalition to find ways to sustain their work, and in doing so, transformed the partnership in to a non-profit agency (outcome^1^). The non-profit agency became a new community resource for a widening array of community health issues (outcome^1^ - > enabling context^2^ in a ripple or reinforcing feedback effect).

The creation of resources and motivation within partnerships led to other project ideas and activities that were not originally intended or planned. These new projects were facilitated by the work already conducted and the motivation of partnership members. For example, a community pastor involved in a partnership was able to start a new project in a different neighbourhood, based on expertise he gained through the initial partnership:

Partnership H – Academic stakeholder *“When the pastor moved to another neighborhood - because we were [already] involved in the partnership together, he saw that there were children there that needed help, so we started [a new] project over there”.*

##### CMO configuration

The partnership had been established for a number of years (context^1^). The community partner, relying on trusting relationships developed with academic members (mechanism^1^), and expertise gained in research (outcome^1a^), identified new areas of need for research beyond original goals (outcome^1b^). Prior partnering ensured that the coalition was research ready and was able to create an unanticipated spin-off project (outcome^1^ - > context^2^).

Spin-off activity was shown to occur for the academic partners as well. The following quotation illustrates how academic professional development was gained and used to redesign a university-based, foundation-sponsored Clinical Scholars program that emphasized CBPR in the training of physician researchers:

Partnership E – Academic stakeholder *“the Director of a [U.S. National] Clinical Scholars program asked me if… I’d redesign the program based on a CBPR perspective. So that was about 7 years ago, so I did that. The scholars program itself has been a very powerful thing going on… my particular edge was on the one hand, dealing with a highly stigmatized set of illnesses, and on the other hand, really working on bringing the partnership into… highly rigorous methods…such as doing a large RCT. And figuring out how to do that by addressing all the trust issues”.*

##### CMO configuration

The academic stakeholder had been working in partnership with community members for many years (context^1^) and though valuing CBPR practice (mechanism^1^), gained expertise in the conduct of ethically sensitive research in partnership with community groups (outcome^1a^). This expertise was seen to have relevance to the director of a national U.S. Clinical Scholars program (outcome^1b^) and curriculum of Robert Wood Johnson Clinical Scholars Program was redesigned based on this expertise, creating a diffusion of CBPR research innovations (outcome^1^ - > context^2^).

##### Systemic transformations

Systemic transformations took the form of cultural shifts, the implementation of new policies and improvement to health service provision. In the following quotation a community partner describes how the academic-community relationship helped to break cultural taboos around the word ‘cancer’. This led to radically new perspectives on the disease, as well as increased prevention and diagnosis:

Partnership D- Community stakeholder. *I’m just in awe about the breakthrough [we had] in cancer awareness in [our community. We didn’t even say the word [cancer], it was culturally taboo…When there was a diagnosis, they went through treatment alone and it was only at the end of their life that they shared this information. And now, it’s like this has all changed due to the impact of cancer awareness to the efforts of [the Partnership]. Now [community] women overcome these cultural barriers and it’s ok. We can talk about the mammogram. We can say the word cancer and we’re not asking for it to come upon us…women are approaching us in public places and asking ‘could you schedule me for a pap test or a mammogram?’ , and that’s the shift. I mean this was unexpected.*

##### CMO configuration

Prior to development of the partnership, the cultural norm was to see cancer as an inevitably terminal illness not to be mentioned except in retrospect at the end of life (context). The development and strengthening of trust between academic and community partners of the coalition (mechanism^1a^) and the trust of the coalition by community members at large (mechanism^1b^) increased awareness of cancer as a treatable condition if diagnosed early, thereby encouraging community members to speak up about cancer screening (outcome^1^). As a result, the stigma and taboo around the disease was reduced overtime (outcome^1^ - > context^2^).

In another example, the systemic change was a change in policy at the state health department level. Here, the partnership lobbied the government to make changes to payment structure to increase universal access to health service:

Partnership C - Academic stakeholder *“the biggest outcome of the entire project was that we got the state health department to change the way that they paid for [cancer] screening. And since they can’t do it for one ethnic group without applying it to everybody [they created a universal policy]”.*

##### CMO configuration

Due to successful research productivity of the partnership (context^1^), the partnership was empowered to advocate for improvements to the way cancer screening was paid for (mechanism^1^). This led to successfully convincing the state health department to change the policy for the community (outcome^1^) which then created a universal policy so it would apply to all people (outcome^1^ - > context^2^).

Finally, systemic transformation was seen in the way community groups were able to realize their self-empowerment. This quotation reveals how community members from a historically colonized population were positively influenced by their participation in CBPR, resulting in new beliefs and actions for community empowerment.

Partnership A – Academic stakeholder *“…the fact that they [community members] became involved in getting control of the hospital in the Northern community was in part a reflection of the increasing activism in the sense that they [realized they] could control their own engagement….I think the partnership aided that process. They understood through the partnership, how you could be in different relationships than they’d been previously with people in authority. So they could assert themselves”.*

##### CMO configuration

The initial partnership dynamics were influenced by the negative history of mistrust (context^1^), but also by the fact that the academic members demonstrated cultural humility, which in part, supported the sense community members’ self-empowerment (mechanism^1^). This self-empowerment led the community to further efforts toward self-determination including gaining control of the community hospital (outcome^1^ - > context^2^).

These findings indicate that CBPR outcomes are complex and require longitudinal examination. Table 2 summarizes the main findings of this research in a composite sketch of CBPR project outcomes using CMO configurations and the ripple effect concept.

**Table 2:** CMO configurations depicting a composite summary of the findings

1. The dynamics and impact of trust building:	
Context^1^	New academic-community relationships were often initiated in a backdrop of community mistrust of the intentions underpinning research, or alternatively, community members may have an overly positive, naïve trust of academics. As members of a partnership began working together, accomplishing early intermediate goals and dealing with conflict and conflict resolution, trust was built and maintained over time.
Mechanism^1^	Perceived trustworthiness of CBPR partnership maintained over the course of time. Trust responses were expressed continually, and were intensified in times of disagreement and conflict. Contextual factors, such as history of oppression and research abuse, and the harsh reality of community health morbidity and mortality, may have triggered trust or mistrust responses. Continually on trial, trust was contingent on how members responded to all circumstances and resources of research.
Outcome^1^ - > Context^2^	
	Trust building, conflict resolution, and trust sustainment over time were intermediary effects that facilitate intended and unintended health improvement outcomes. Trust enabled the sustainment of efforts, new spin-off projects, and systemic change. What was achieved after years of trust-building was done with much less effort and resources when compared to outcomes from early stages of partnerships with little or no trust.
2. Spin-off projects and systemic transformations	
Context^1^	Academic and community members formed partnerships, which transformed into long-term working relationships; over time they became experts in applying research methods and fundraising for complex community health needs.
Mechanism^1^	Partnership stakeholders felt inspired to work on unrelated projects, while relying on expertise and research savvy gained in the former experiences as well as in developing relationships with other community and academic members. In the process of partnering, community members may have gained a sense of empowerment and an appreciation of the value of research and evaluation; through interaction with academics, community members identifies unhealthy cultural taboos, academics gained insight into community strengths and experiences of vulnerability and oppression; expertise was realized in the co-production of ethically sensitive research in complex community contexts.
Outcome^1^ - > Context^2^	
	New appreciation of research and evaluation by community members and health service organizations led to the use of newly acquired research skills in community service delivery -- opening the door to improving the way community services were developed and evaluated. Community organizations were transformed from service delivery entities to community-based research resources, thus transferring research expertise from universities to communities. Communities, through a realization of self-empowerment took actions to improve local conditions and infrastructure. Communities broke taboos that were impeding health promoting behaviors. Academic members used their university positions to spread their gained expertise back to the university, promoting the impact of community strengths and engagement on research, knowledge production, spin-off products and systemic transformations.

## Discussion

In this study, we used realist methodology [24] and middle-range theory [29] to facilitate the assessment of complex CBPR outcomes and showed how partnerships create a host of benefits to individuals, communities, institutions and policy development. We advanced the theory of partnership synergy by studying how trust building and maintenance was a key factor in increasing outputs and sustainability efforts over time, leading to partnership longevity, spin-off projects and systemic transformations. The ‘ripple effect’ concept as another component of the MRT supported the assessment of outcomes, by showing how one effect can lead to another over a course of partnership phases.

Our sample consisted of academic and community stakeholders from partnerships in the USA and Canada built on strong foundations of equity in co-governance and shared decision-making [20]. The effects of such partnership formation and development are significant, considering the challenges in overcoming logistical, cultural and structural obstacles to community health improvement. Reforming health services, enhancing educational curricula, overcoming cultural taboos, creating non-profit societies, and increased empowerment and pro-action against morbidity are all important CBPR outcomes shown in this research. Thus investment in research partnerships may have significant returns which are unforeseeable at the outset, and require evaluation using complexity sensitive methodology such as realist evaluation.

The research presented here can be situated within a broader literature of understanding the science of CBPR, in which Wallerstein and colleagues’ work on CBPR modelling and assessment is perhaps the most relevant [8, 9, 12, 14, 18]. Their conceptual model of CBPR is similar to the way we have theorized participation including the need to understand context, trust, and relational dynamics in the production of complex outcomes. It is possible that realist evaluation in tandem with other approaches can enhance CBPR assessments [7, 8, 12], implementation [34, 35], policies development [14] and evidence integration [36], as many of these efforts recognize trust issues as central to CBPR frameworks. Specifically, qualitative studies can inform CBPR assessments by identifying the pathways (CMOs) that can be tested deductively using measures such as identified by Oetzel et al [9] and providing insight into the selection of the most appropriate measures to test these pathways.

An innovative aspect of this study was the sequential application of the realist approach (*i.e.*, realist review of published CBPR studies followed by interviews with the authors of these studies, with realist analysis of the new empirical data) leading to cumulative theory development in the area of CBPR. Our finding of trust building and maintenance over time, partnership synergy of academic- community partnerships and intended and unanticipated benefits to the community, contrasts somewhat with the current pressure on research scholars in public health and beyond to demonstrate and measure how research leads to ‘impact’. Responses to such pressures are sometimes couched in simplistic and linear terms, depicting communities as (more or less) the passive recipients of research findings [37]. Here we present a detailed theory of CBPR impact, which suggests that equitable co-governance based on trust may (in the right contextual conditions) lead to sustained partnerships, resulting in the generation of effective solutions to the complex problems of public health. From that perspective, our findings have significant implications for improving the effectiveness of programs, long-term sustainability, health change motivation, and finding potential solutions to the complex array of cultural, logistical and economic barriers to affecting change in many community-based settings.

Our findings on the reinforcement of trust over time in CBPR can be conceptualized with reference to the critical social science literature on trust and power. Greener, drawing on Lukes’ writings on the ‘three faces of power’ [38], distinguishes three kinds of trust in a healthcare setting: voluntary trust (achieved through close interpersonal relationships built over time, and defined as “a consensual absence of calculation, where we voluntarily forego calculating in a relationship”), involuntary trust (born of enforced dependency, for example trusting doctors when one is perilously ill), and hegemonic trust (when “we trust without realising there is an alternative”) [39]. The trust relationships in CBPR are typically voluntary and based on a personal sense of commonality and reciprocity. Individuals from disempowered and marginalised groups have been shown to have low levels of trust in conventional health services, and that trust may be enhanced through interpersonal relationships and continuity of care [40].

### Limitations

Our sample consisted of community-academic research partnerships that exhibited high levels of co-decision-making and co-governance over time [20]. We did not interview, and therefore can draw no conclusions about, partnerships that broke up or (for whatever reason) declined to participate. Thus whilst the findings from the successful partnerships included in this study are consistent and credible, we cannot extrapolate from them to draw conclusions about the mechanisms operating in the converse situation (*e.g.*, when co-decision making is weak or trust is not built). In acquiring the sample for the study, we contacted academic authors of published CBPR studies and asked them to recommend community partners for interviewing. Thus we had to rely on the recommendation of the academic partners, which may have created a particular bias in the sample. As well, fewer numbers of community members were available for interviews as compared to academic members, which also created bias in the sample and is reflected in the analysis. Another limitation is that roughly half of the interviews were conducted by phone or skype which was a necessary limitation due to travel funding constraints.

What has not been covered by our research is examining how and when trust building fails, and the contextual factors involved in the failure of research partnerships to achieve their goals. Indeed, there is work to be done studying community-academic relationships that were not sustained over time, to examine the extent to which failure of both building and maintaining trust leads to the collapse of partnership. In such situations, outcomes could include deepening of mistrust between communities and outsiders. In addition, we caution that a top-down notion of public health sustainability is contentious [41]. Budget cuts, shifting government priorities and volunteer burnout are critical factors in defining the nature of sustainable efforts which have not been fully explored in the current research. This will need to be theorized to inform a comprehensive view of how CBPR can contribute to the public health agenda.

## Conclusion

Evidence-informed theorizing about how and in what circumstances CBPR works should be an on-going pursuit. Here we have provided theory and evidence showing that complex health improvement efforts can be addressed by a partnership approach involving shared decision making and equitable co-governance across the stages of research. We have indicated that trust building and maintenance can make significant contributions to sustainability and systemic transformation which are key to both increasing the knowledge of factors supporting successful community-academic partnerships and transforming contexts to improve the conditions and motivations that determine health status. We hope that this research, illuminating the complex requirements for health improvement and the role of CBPR therein, can inspire new ideas for sustainable approaches to public health and related fields.

## Additional files

Additional file 1:
**Interviewee table.**
Additional file 2:
**Interview guide.**
