# Supplementary material for: A realist evaluation of community-based participatory research: partnership synergy, trust building and related ripple effects
Source: BMC Public Health. 2015 Jul 30;15:725. doi: 10.1186/s12889-015-1949-1 (PMC4520009; doi:10.1186/s12889-015-1949-1)
Supplement: Additional file 1: — Interviewee table. [file 12889_2015_1949_MOESM1_ESM.docx]

Appendix 1: Interviewee Table

| Partnership | # academic members interviewed | #community members interviewed | Separate interviews or focus group | Interviewed by Phone or In person? |
| --- | --- | --- | --- | --- |
| A | 1 | 0 | n/a | In person |
| B | 1 | 1 | Separate | In person |
| C | 1 | 1 | separate | Phone |
| D | 1 | 1 | Separate | In person & Phone |
| E | 2 | 1 | Separate | In person & Phone |
| F | 1 | 0 | n/a | Phone |
| G | 2 | 1 | Academics together, community separate | In person |
| H | 1 | 4 | Combined focus group | In person |
| I | 2 | 1 | 1 Academic and 1 community combined + 1 one separate academic | In person & Phone |
| J | 1 | 0 | n/a | In person |
| K | 1 | 0 | n/a | Phone |
| Total | 14 | 10 |  |  |
