# Supplementary material for: A realist evaluation of community-based participatory research: partnership synergy, trust building and related ripple effects
Source: BMC Public Health. 2015 Jul 30;15:725. doi: 10.1186/s12889-015-1949-1 (PMC4520009; doi:10.1186/s12889-015-1949-1)
Supplement: Additional file 2: — Interview guide. [file 12889_2015_1949_MOESM2_ESM.docx]

Appendix 2: Interview Guide

**PROJECT TITLE: Toward Key Principles for Assessing Participatory Research and Integrated Knowledge Translation**

Interviewer name: Justin Jagosh

This interview guide is a series of open-ended questions based on the research objectives (see below). The interviews are intended to be a conversation between two people and the questions presented here serve to guide the interview and ensure that the dialogue covers all relevant areas.

Interviews will begin with introductions and a brief overview of the participants’ involvement in the partnership.

Objectives: (a) to ascertain how coalition stakeholder describe PR; (b) to receive their input on the findings of our realist review; (c) to fill in the knowledge gaps from the limitations of the literature-based realist review

Participants will be encouraged to read and ask questions about the consent form. Once the consent form is signed, they will be asked if they have any other questions before the interview begins.

Section I: Introduction

- Could you begin by describing your role in the partnership?
- How would you briefly describe the activities of the partnership?

Section II: Understanding the pre-existing factors (pre-dating the partnership formation) which impact the nature of the partnership and outcomes produced

- Describe, in your terms, elements of the pre-existing context (community and academic settings for example), before research partnership formation
- What impact does community cohesiveness, community readiness, geographic location of community setting have on the outcomes of partnering?
- What impact does academic training, academic setting (institutional policies etc.) have on partnership outcomes?
- What impact do larger political, economic, and social factors have on partnership outcomes? Examples:

Section III: Understanding the generating factors which create partnership outcomes

In our systematic realist review, we identified numerous ways in which PR generated outcomes to research and intervention programming. (Then show list of demi-regularities).

- Does your experience resonate with our overall findings summarized through the following statements:

Demi-regularity 1 (Hypothesis b):

1. **PR generates culturally and logistically appropriate research characteristics related to:**

1.1 Shaping the scope and direction of research

1.2 Developing program and research protocols

1.3 Implementing program and research protocols

1.4 Interpreting and disseminating research findings

Demi-regularity 2 (Hypothesis b):

2. **PR generates recruitment capacity of:**

2.1 community members to the advisory board

2.2 community members for implementation (specifically for lay health worker programs)

2.3 community members as recipients of programs (intervention enrolment)

Demi-regularity 3 (Hypothesis b):

**3. PR generates capacity of:**

3.1 the community partners

3.2 the research partners

Demi-regularity 4 (Hypothesis b):

**4. PR generates disagreements between the co-governing stakeholders during decision-making processes,** resulting in both:

4.1 positive outcomes for subsequent programming

4.2 negative outcomes for subsequent programming

Demi-regularity 5 (Hypothesis c):

**5. PR synergy accumulates in cases of repeated successful outcomes in partnering, thus increasing the quality of outputs and outcomes over time**

Demi-regularity 6 (hypothesis c):

6. Partnership synergy accumulates capacity to sustain project goals beyond funded timeframes and during gaps in external funding

Demi-regularity 7 (hypothesis c):

7. PR generates systemic changes and new unanticipated projects and activity

Section IV: Understanding the cumulating factors which create partnership outcomes:

- Our review showed that in some cases, the partnership was able to generate spin-off projects due to the momentum gained and numerous successes in partnering. Has this also been your experience?
- Would you say that partnering, if successful, builds momentum over time, such that the outcomes of one phase of research impact subsequent phases? (also that the collaboration changes the context over time).

Section V: Conflict and Differences of Opinion

- Almost all our partnerships described instances of conflict or differing world views among co-governing stakeholders. Can you identify examples of the causes of conflict? What effect does conflict and negotiation play in generating outcomes of the collaborative process?
